# Prevalence, clinical presentations, associated risk factors and recurrence of laryngeal papillomatosis among inpatients attended at a Tertiary Hospital in Northern zone Tanzania

**DOI:** 10.11604/pamj.2018.30.209.11211

**Published:** 2018-07-16

**Authors:** Marco James, Denis Katundu, Desderius Chussi, Peter Shija

**Affiliations:** 1 Kilimanjaro Christian Medical University College, Moshi-Tanzania; 2 Department of ENT, Kilimanjaro Christian Medical Center, Moshi-Tanzania

**Keywords:** Laryngeal-papillomatosis, risk, factors, clinical, features, recurrence

## Abstract

**Introduction:**

Although Laryngeal papillomatosis is a rare disease and can be conventionally managed through surgical excision as well as adjuvant therapy yet Laryngeal papillomatosis has high tendency to recur raising its prevalence in the community, airway involvement warrants dangerous complications requiring emergency tracheostomy, especially if clinical course is poorly understood and misdiagnosis is common. The study aims to determine the prevalence, clinical features, risk factors and recurrence of Laryngeal Papillomatosis among patients attending ENT department at KCMC from 2005 to 2015.

**Methods:**

This was a 10 year experience/ Descriptive hospital based cross-sectional study conducted based on patients' medical record at KCMC. Information was recorded into data collection sheets, entered and analyzed through SPSS version 20, summarized and presented in tables and charts, proportions and percentage used to compare groups.

**Results:**

51 patients were identified over the 10 years study period corresponding to a prevalence of 0.09%. 26 were males and 25 females, ages ranging from 1 to 67 years. The median age of onset was 6 years. Children less than 5 years accounted for 19 (37.3%) and 58.8% had repeated surgeries. Clinical presentation ranged from hoarseness in 43 (84.3%) patients to weight loss 3 (5.9%) and 96.1% showed complete or partial presence of the clinical triad of risk factors.

**Conclusion:**

Laryngeal papillomatosis is a rare disease that carries severe morbidity due to its highly recurring nature. It is primarily a paediatric disease, generally affecting more males; its clinical features highly resemble other airway obstructive diseases and therefore a careful thorough clinical evaluation is required in order to correctly diagnose Laryngeal papillomatosis.

## Introduction

Laryngeal Papillomatosis (LP) is benign disease of the airway mucosa characterized by the development of exophytic proliferative lesions of connective tissue covered by epithelium due to HPV infection. It is termed laryngeal papillomatosis or glottal papillomatosis due to the strong predilection for the larynx but papillomas may present anywhere along the respiratory tract [1-3]. Although laryngeal papillomatosis is a rare disease [4] its highly recurrent nature and involvement of the airways upturns its burden to patients and the community [5,6] and it is slightly more prevalent in males [3,4,7-9]. As a result of airway obstruction, laryngeal papillomatosis is commonly misdiagnosed as asthma, bronchitis, laryngeal nodule or croup leading to late diagnosis and acute airway complications [10,11], of which some requiring tracheotomy for immediate breathing and further airway management [12]. While diagnosis is based on the clinical symptoms, nearly 100% of patients present with hoarseness followed by dyspnea [3,4], however this is not always as some patients may not present with hoarseness [7] and others present in a variety of combinations of clinical feature [13]. A clear understanding of the clinical course and features is thus required in order to distinguish it from other airway obstructive diseases.

The clinical triad of a first born delivered vaginally to a young (teenage) mother has been previously noted among juvenile onset laryngeal papillomatosis patients (JO-LP) [14]. A complete or partial triad was observed in 72% of JO-LP patients, 36% of adult on set LP (AO-RRP) patients, 29% of juvenile controls, and 38% of adult controls; among adult participants, patients reported more lifetime sex partners and a higher frequency of oral sex than reported by adult controls [14,15]. However, more survey of the presence of such important clinical aspects especially in developing countries such as Tanzania is needed. Recurrence of laryngeal papillomatosis is reported differently by different studies with the highest being observed in Denmark (73.7%) [16] And lowest in Nigeria 6.9% [3]. Up to date laryngeal papillomatosis is still misdiagnosed as asthma, croup, or chronic bronchitis because they all display symptoms of airway obstruction. However, as laryngeal papillomatosis involves respiratory system misdiagnosis or delayed diagnosis will predispose to emergency airway obstruction or longstanding complications due to inadequate gaseous exchange [17]. Most of the reviewed literatures from countries other than Tanzania seem to underestimate the prevalence of LP when applied to the northern zone of Tanzania, as they report the prevalence to be in a range of 0.002% [3] to 0.005% [7] whereas KCMC receive an approximate of more than 15 patients per year; these differences may impair the correct planning for health needs as far as LP is concerned. The aim of the study was to determine the prevalence, clinical presentations and recurrence, and describe the clinical triad of risk factors for laryngeal papillomatosis among patients attending ENT department at Kilimanjaro Christian Medical Center from 2005 to 2015.

## Methods

### Study design and setting

A descriptive hospital based cross-sectional study conducted based on patients' medical records from 2005 to 2015 at the department of ear, nose and throat (ENT) of the Kilimanjaro Christian Medical Center (KCMC), a tertiary referral hospital in northern Tanzania.

### Study population

The study included all patients who attended and treated in the ENT department at KCMC from January 2005 to December 2015 with complete registry information; this includes both the newly diagnosed, follow up and recurrent cases within the study period. Patients whose diagnosis is unknown or not clear, as well as Patients with coexisting laryngeal malignance were also excluded from the study. At the end the study enrolled a sample of 51 laryngeal papillomatosis patients.

### Variables

The main outcome of interest in this study was Laryngeal papillomatosis while the independent variables included; Age of the patient, sex, number of revision surgeries (which is the measure of recurrence of the LP), maternal gravidity, maternal age and mode of delivery.

### Data sources

This study utilized theatre registry book from the ENT operating theatre to identify patients surgically managed for laryngeal papillomatosis, then patients' files were traced at the Medical record department for retrieval of the required information.

### Data collection

Descriptive information such as patients' age at diagnosis, sex, number of surgeries, maternal age, gravidity and mode of delivery were collected through review of patients' files in the medical record department of KCMC consultant hospital using data extraction sheet in which all required particulars were filled in and then transferred into a data set.

### Ethical clearance

The ethical approval was obtained from the Tumaini University Makumira KCMUCo Ethical Research Committee Board; permission to use theatre registry books was obtained from the head of the ENT department while permission to use patients' files was granted from the head of the medical record department. Furthermore, no patients name or file number was used, instead participants' codes were assigned for patients' confidentiality.

### Data processing and analysis

Data were analyzed using statistical package for social sciences (SPSS) version 20.0 (SPSS Inc. Chicago, III). Categorical variables such as sex, mode of delivery was summarized using proportions, percentages while continues variables such as age, recurrence were summarized using mean and standard deviation (for normal distribution), and median and range (for skewed distribution), statistics are presented into frequency distribution tables and charts.

## Results

### Socio-demographic characteristics of the study participants

The socio-demographic characteristics of the study participants are summarized in the Table 1. A total of 51 cases of histologically confirmed LP were identified. The age of the study participants ranged from 1year to 67 years; with the median age of onset at 6 years (IQR=8.5) and mode of 2 years. Children less than 5 years of age had the highest prevalence 19 (37.3%). Males were slightly more affected 26 (51%). Majority of the patients were pre-school children 27 (53%) and primary school pupils 16 (31.3%).

**Table 1:** Socio-demographic characteristics of inpatients with LP in the ENT department at KCMC (N= 51)

Characteristicts		N(%)
**Age (years)**	0-4	19 (37.3)
	5-9	17 (33.3)
	10-14	7 (13.7)
	15-19	1 (2)
	≥20	7 (13.7)
**Median age**	6	
**Range**	67	
**Sex**		
	Male	26 (51)
	Female	25 (49)
**Occupation**	Children	27 (53)
	Pupils	16 (31.3)
	Students	2 (3.9)
	Peasants	3 (5.9)
	Business	3 (5.9)

### Prevalence of laryngeal papillomatosis by demography

**Figure 1:**
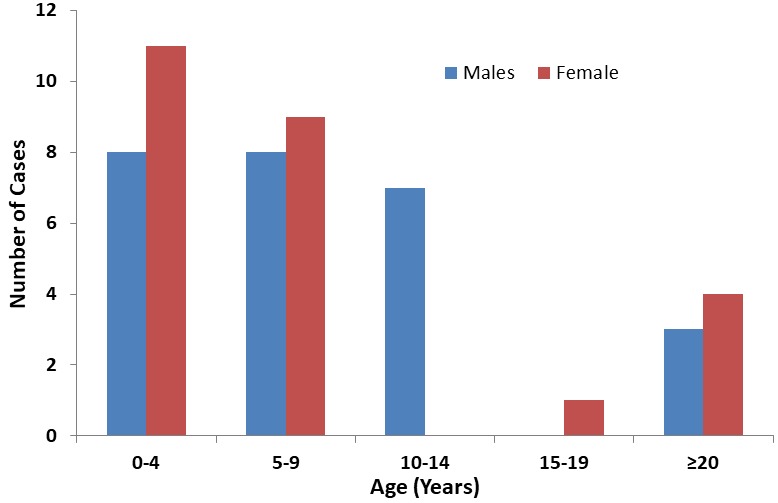
Distribution of LP cases by age and sex at KCMC, ENT department

During the 10-year period this study looked at, there were 56,510 patients attended at the ENT department out of whom 51 cases were of LP corresponding to a prevalence of 0.09%. Among the 51case of LP, Juvenile onset LP had the highest prevalence 44 (86.3%) (Table 1). Of the JOLP, under 5years of age had the highest prevalence 19(37.3%) while the adolescent group (15-19years) had the lowest prevalence 1(2%) (Table 1) Females were more than males for patient 0-9years of age and ≥ 20 years while for patients 10-14 years the disease is entirely observed in males (Figure 1).

### Clinical presentations of laryngeal papillomatosis

In this study population hoarseness of voice was the most commonly seen sign in 43 (84.3%) patients, followed by difficulty in breathing (DIB) 35 (68.3%), decrease/loss of voice 23 (45.1%), cough 9 (17.6), inspiratory stridor 8 (15.7%), and the less common presentations were snoring 4 (7.8%), mouth breathing 2 (3.9%), foreign body (FB) sensation 1 (2%) and weight loss 3 (5.9%). Majority of cases 78.4% presented with a combination of clinical features (Table 2) while the rest presented with isolated symptoms of hoarseness of voice 9 (17.6%), loss of voice 1 (2%) and DIB 1 (2%); Snoring, mouth breathing and weight loss were entirely seen in the younger population while foreign body sensation, was prevalent in the adult population.

**Table 2:** Pattern of clinical presentations of LP patients during diagnosis, attended in the ENT department at KCMC (N = 51)

Clinical pattern	Frequency	Percentage
Hoarseness	9	17.6
DIB	1	2
Loss of voice	1	2
Hoarseness+ other features	35	68.6
DIB+other features	5	9.8
**Total**	**51**	**100**

### The clinical triad of risk factors for laryngeal papillomatosis

**Figure 2:**
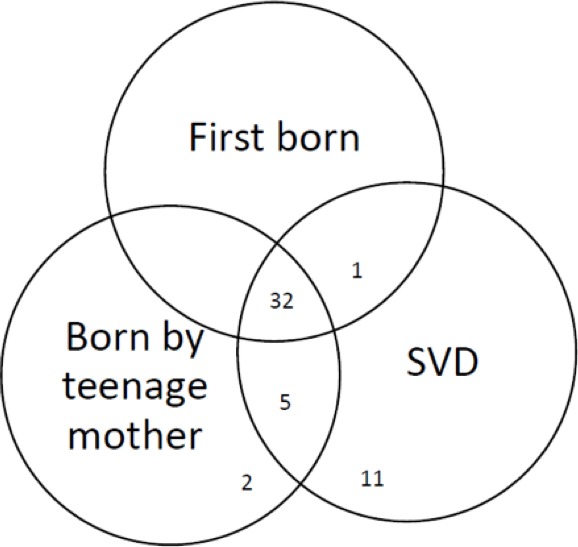
Clinical triad of risk factors for LP among 51 patients at KCMC, ENT department

32 (62.8%) patient had a complete triad of risk factors (first born of a teenage mother delivered by SVD) and between 64% and 96% had at least one component of the triad (Figure 2).

### Number of revision surgeries of patients treated for laryngeal papillomatosis

**Figure 3:**
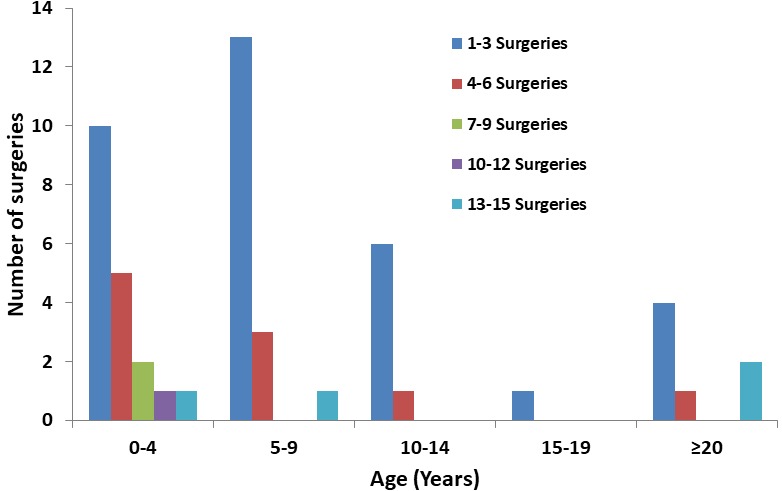
Number of surgeries in different age groups, KCMC, ENT department

The number of surgeries among patients with LP varied over a wide range, from one surgery in 21(41.2%) cases to 15 surgeries in 2 (3.9%) cases; the median number of surgeries was 2 during this 10-year period of observation; 30 cases had recurrent episodes following microlaryngeal surgery (MLS) making the recurrence rate 58.8%. The highest number of surgeries (15) was among patients above 20 years of age; while the highest number of surgeries observed among JOLP was 13 surgeries in two cases aged 9 and 13 years (Figure 3).

## Discussion

The prevalence of LP among patients attending ENT department at KCMC hospital is as high as 12-30 folds (0.09%) than that is seen in developed countries such as that has been shown by studies done in the USA which showed a prevalent range between 0.002% in adults and 0.004% in children and in Europe an average of 3 to 7cases per million population [5]. This could probably due to poor management and screening tendencies of women for cervical papilloma in Tanzania; such difference could actually underestimate the burden of LP and so affects allocation of resources for its preventive and management measures. Previous studies have shown that LP in most cases is affecting a paediatric population and the highest prevalence is among under 5 years [1,4,5]. Similar observations were found in our study in which under 5 year patients had the highest prevalence while the adolescent age group (15-19 years) had the lowest prevalence 1 (2%), this can possibly be due to the fact that laryngeal papillomatosis tends to subside at this age or upon attaining puberty. Although most of the studies show males to be more affected [3,4,7,8], our study showed that females are more affected 11 (57.9%) than males 8(42.1%) for patients under 5years and in AOLP in which females were 4 (57.1%) while males 3 (42.9%), other age groups agree with findings from the previous studies. However, generally males have high prevalence in JOLP as well as AOLP. Although the commonest presenting features are hoarseness and airway obstruction, only 43 (84.6%) cases presented with hoarseness in contrast to most of other studies in which all patients (100%) studied presented with hoarseness [3,4,13]. In conformity with a previous study in Senegal [13] majority of cases presented with variety of clinical features, rather than isolated features and so resembling other airway obstructive diseases such as asthma, laryngotracheobronchitis, chronic bronchitis, laryngeal nodules and gastro-esophageal reflux diseases, hence can be easily misdiagnosed for LP; since some patients with LP do not present with hoarseness of voice as it is anticipated by most of medical practitioners, its diagnosis can be easily missed and so delays prompt management and subsequent airway complications.

The occurrence of complete or incomplete clinical triad of risk factors in our study was apparently higher (96.1%) than that seen at Johns Hopkins medical center, USA (72%) [14], and was mostly seen in JOLP. This can possibly be explained by poor screening and vaccination tendency against HPV among pregnant women in developing countries including Tanzania. Therefore, pregnant women should be careful examined for sexually transmitted infections (STI) related symptoms, especially presence of genital warts in order to promptly plan preventive measures as far as LP is concerned. Since up to 96.1% are born through spontaneous vaginal delivery (SVD), cesarean delivery should be discussed as the option in expectant mothers with condylomas since they are at a higher risk for transmission of JOLP to their infants [18]. Laryngeal papillomatosis is highly characterized by recurrence even after surgical excision of the lesions; although the rate of recurrence of LP in this study is less (58.8%) than that observed in Denmark in a 20-year period of observation (73.7%), the author of that study suggested that the presence of maternal history of condyloma had an impact on the recurrence rate of laryngeal papillomatosis [17]; nevertheless, recurrence rate observed in this current study is higher than that seen in studies done in Nigeria (6.9%) [3] and Dakar, Senegal (26.2%) [13]. However, the number of surgeries in this current study can be underestimated due to poor documentation and attendance of the patients to other hospital in some episodes. Further studies to explore the association between the observed risk factors and the occurrence of LP and factors associated with its recurrence nature and management need to be done in the future.

## Conclusion

Laryngeal papillomatosis is a disease that carries severe morbidity as its clinical course is characterized by a highly recurring nature. It is primarily a paediatric disease and generally affects more males than females; its clinical features highly resemble other airway obstructive diseases and therefore a careful thorough clinical evaluation is required in order correctly diagnose it. Also, public health education is thus crucial in identification of the early presenting signs and symptoms suspicious to Laryngeal papillomatosis.

### What is known about this topic

- LP is more prevalent in males than females;
- Hoarseness is a hallmark for diagnosis of LP.

### What this study adds

- LP have different sex prevalence in different age group;
- LP presents in most cases in a combination of clinical symptoms rather than isolated hoarseness;
- Some patients do not present with hoarseness.

## Competing interests

The authors declare no competing interest.
